# Anterior gastric wall anastomosis may lead to lower rate of delayed gastric emptying after minimally invasive Ivor Lewis esophagectomy: a retrospective cohort study

**DOI:** 10.1007/s00464-024-10696-z

**Published:** 2024-02-09

**Authors:** Eren Uzun, Alberto d’Amore, Felix Berlth, Carolina Mann, Evangelos Tagkalos, Edin Hadzijusufovic, Hauke Lang, Peter Philipp Grimminger

**Affiliations:** Department of General-, Visceral- and Transplant Surgery, University Medical Center of the Johannes Gutenberg University, Langenbeckstrasse 1, 55131 Mainz, Germany

**Keywords:** Esophageal cancer, Minimally invasive, MIE, RAMIE, Anastomosis

## Abstract

**Introduction:**

In minimally invasive esophagectomy, a circular stapled anastomosis is common, but no evidence exists investigating the role of the specific localization of the anastomosis. The aim of this study is to evaluate the impact of an esophagogastrostomy on the anterior or posterior wall of the gastric conduit on the postoperative outcomes.

**Material and methods:**

All oncologic minimally invasive Ivor Lewis procedures, performed between 2017 and 2022, were included in this study. The cohort was divided in two groups: a) intrathoracic esophagogastrostomy on the anterior gastric wall of the conduit (ANT, *n* = 285, 65%) and b) on the posterior gastric wall (POST, *n* = 154, 35%). Clinicopathological parameters and short-term outcomes were compared between both groups by retrieving data from the prospective database.

**Results:**

Overall, 439 patients were included, baseline characteristics were similar in both groups, there was a higher proportion of squamous cell carcinoma in ANT (22.8% vs. 16.2%, *P* = 0.043). A higher rate of robotic-assisted procedures was observed in ANT (71.2% vs. 49.4%). Anastomotic leakage rate was similar in both groups (ANT 10.4% vs. POST 9.8%, *P* = 0.851). Overall complication rate and Clavien–Dindo > 3 complication rates were higher in POST compared to ANT: 53.2% vs. 40% (*P* = 0.008) and 36.9% vs. 25.7% (*P* = 0.014), respectively. The rate of delayed gastric emptying (20.1% vs. 7.4%, *P* < 0.001) and nosocomial pneumonia (22.1% vs. 14.8%, *P* = 0.05) was significantly higher in POST.

**Conclusion:**

Patients undergoing minimally invasive Ivor Lewis esophagectomy with an intrathoracic circular stapled anastomosis may benefit from esophagogastrostomy on the anterior wall of the gastric conduit, in terms of lower rate of delayed gastric emptying.

Totally minimally invasive Ivor Lewis (TMIIL) esophagectomy, either carried out laparoscopically and thoracoscopically (MIE) or robot-assisted (RAMIE), has gained wide consensus because of substantial benefits in comparison to conventional open procedures [1–3]. Particularly, less postoperative (cardiopulmonary) complications, less postoperative pain, and shorter intensive care unit (ICU) stay [4–6] were noticed. Despite the advantages, MIE and RAMIE are a technically demanding procedure and the optimal surgical technique is not yet standardized. Concerning the anastomosis, several techniques were described: hand-sewn, linear stapled, and circular stapled anastomosis [7–9]. Until now, no superiority of one technique over the others arose [10, 11]. Our team performs both MIE and RAMIE with a technically identical circular stapled esophagogastric anastomosis to restore upper gastrointestinal continuity [9]. This technique presents a huge number of possible variants, particularly referring to anvil positioning in the proximal esophageal stump (e.g., transthoracic vs. transoral). Whether among the same technique, different variants could lead to different outcomes is still unclear.

To avoid tension or rotation of the gastric conduit, it is important to determine the best suitable location for the anastomosis. Both anterior and posterior gastric wall could be used to construct the anastomosis and no data in literature suggest preferring one or the other. The aim of the study was to determine whether the specific location (anterior vs. posterior gastric wall) of a circular stapled esophagogastric anastomosis could affect postoperative outcomes. 

## Materials and methods

### Study protocol

All procedures were conducted in accordance with the ethical standards and with the Helsinki Declaration. This study was registered retrospectively (2020-15309-retrospektiv) Informed consent was not required due to the retrospective design of the study. Ethical approval is not needed. Due to the retrospective observational type of the study, there is no patient or public involvement in this research between January 2017 and September 2022, 439 consecutive patients, with lower esophageal cancer or Siewert type 1 or 2 gastroesophageal junction (GEJ) carcinoma, underwent a TMIIL, and were enrolled in this single-center retrospective cohort study. All procedures were performed in a German tertiary high-volume center for cancer. From this cohort, 285 (65%) patients received an anterior wall stapled anastomosis (ANT group), whereas 154 (35%) a posterior wall stapled anastomosis (POST group), operated by the same surgeon.

Patients’ data (including demographic characteristics, comorbidities, preoperative staging and neoadjuvant treatment, intraoperative details, and postoperative outcomes) were prospectively collected and retrospectively analyzed. Postoperative complications were defined according to the modified Clavien–Dindo (CD) classification [12]. Inclusion criteria were resectable esophageal and junctional cancer who underwent a TMIIL with a circular stapled anastomosis. Exclusion criteria were different type of esophagectomies other than TMIIL, conversion to an open approach, and other anastomotic techniques. For detailed information see Flowchart presented in Fig. 3. The primary endpoint was to compare anastomotic leak (AL) rate between the two groups. The secondary endpoints were all the other postoperative short-term outcomes (e.g., delayed gastric emptying, pneumonia, hospital/ICU stay, mortality.) 

### Statistical analysis

Statistical analysis was performed using SPSS version 29.0 (SPSS, Chicago, IL, USA). All continuous data are presented as medians with range (minimum and maximum) or means with standard deviations. Results for categorical variables are presented as numbers with corresponding percentages. To evaluate significance of differences between groups, the Mann–Whitney *U* test was used for continuous variables and the chi-squared test was used as for categorical variables.

### Preoperative protocol

All patients underwent a standard preoperative work-up, according to the current guidelines of the German Cancer Society (DKG), including a gastroscopy with biopsy, blood tests, and a CT scan with contrast enhancement of the abdomen and the chest. All clinical cases were discussed in the multidisciplinary tumor board to define the most appropriate path of care. Each patient underwent a gastroscopy where the pylorus was dilated for 2 min using a 20-mm controlled radial expansion (CRE) esophageal balloon dilatator (Boston Scientific, Ireland). The dilatation was performed through the scope (TTS) under endoscopic vision by inflating water in the CRE balloon dilatator with a maximum inflation pressure of 608 kPa [13].

### Operative intervention

All patients underwent Ivor Lewis (IL) esophagectomy, with loco-regional lymphadenectomy and gastric conduit reconstruction. Both minimally invasive esophagectomies (MIE) and robot-assisted minimally invasive esophagectomies (RAMIE) were performed. The Da Vinci® Xi robotic system (Intuitive Surgical Inc., Sunnyvale, CA, USA) was used for the RAMIE procedure.

In the abdominal phase, the patient lays in a supine, anti-Trendelenburg position. It mainly consists in gastrolysis, forming the gastric conduit and regional lymph node dissection (D1 and D2 lymphadenectomy).

**Fig. 1:**
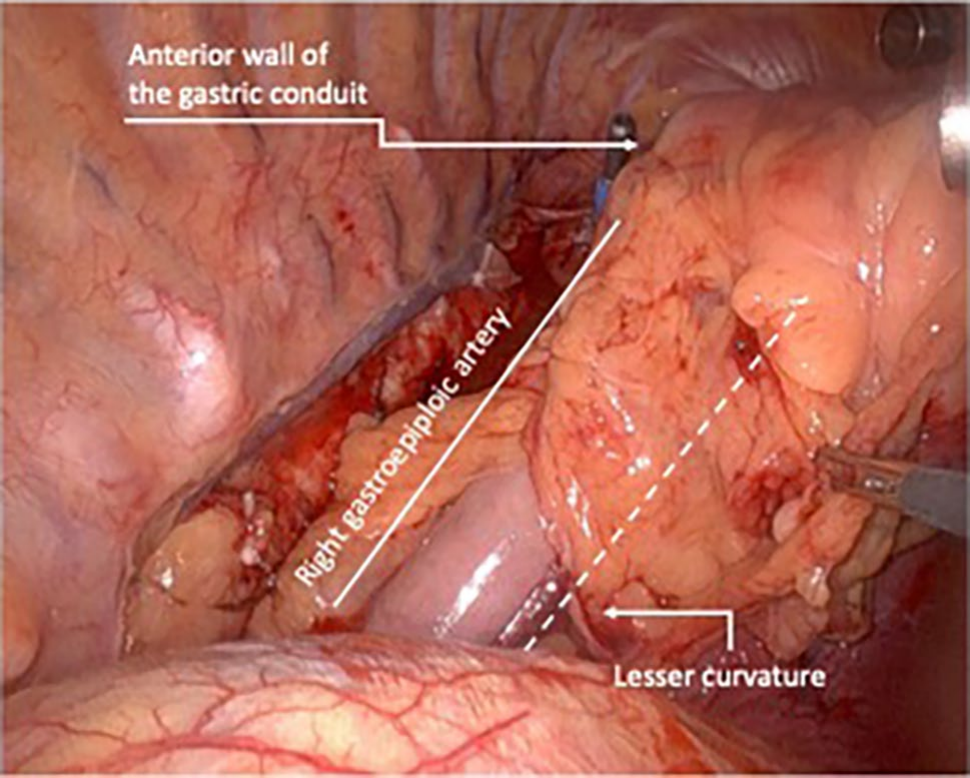
Image showing the anterior wall stapled anastomosis

**Fig. 2:**
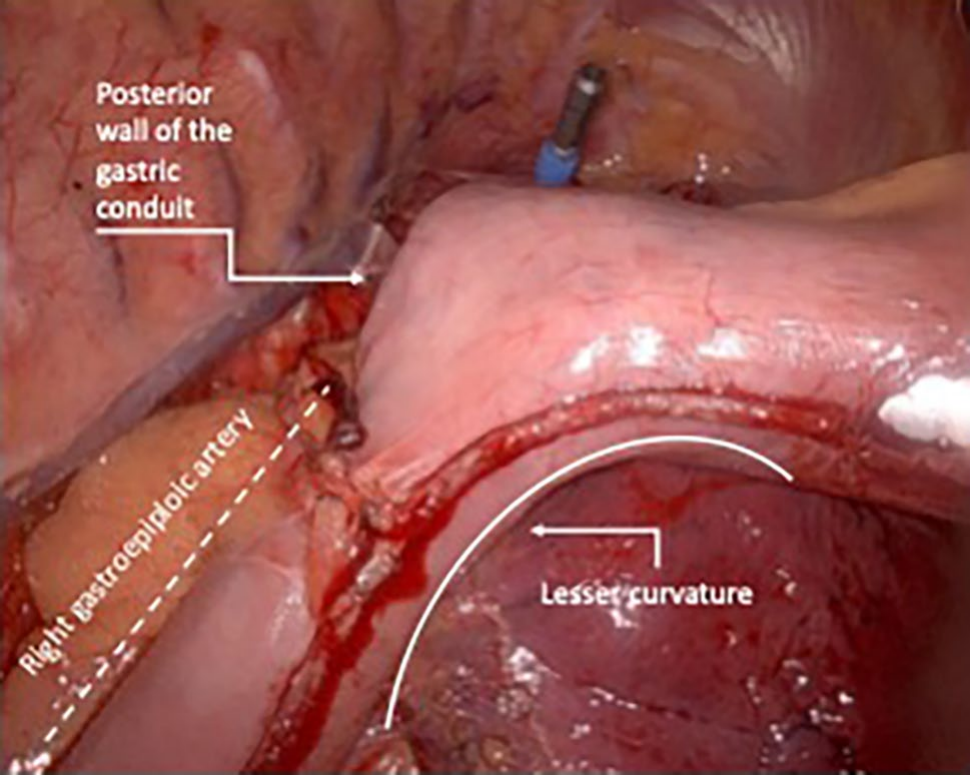
Image showing the posterior wall stapled anastomosis

After the abdominal phase is completed, the patient is turned in a semi-prone position for the thoracic phase. The esophagus is dissected and para-esophageal, lower and middle mediastinal, subcarinal, and paratracheal lymph nodes are harvested. Once the esophagus is completely mobilized, the specimen is transacted above the azygos vein (or higher if needed for oncologic reasons). A purse string suture is tailored on the proximal esophageal stump. Throughout a mini-thoracotomy, the circular staple anvil is inserted into the esophageal stump. To secure the anvil, a second purse string suture is placed. The prepared gastric conduit is gently pulled through the hiatus into the thorax and then brought outside through the mini-thoracotomy. The already transacted lesser curvature of the stomach is opened to insert the circular stapler. At this point, accordingly to the aspect of the arranged gastric conduit, the operating surgeon decides due to anatomy whether to create the anastomosis on the anterior or on the posterior wall of the stomach to construct a tensionless, not twisted esophagogastrostomy. Figure 1 shows the positioning of the stapler for an anterior wall anastomosis, whereas Fig. 2 shows the anastomosis on the posterior wall. The anastomosis is created in an end-to-side fashion and the remnant gastric tube is stapled off with a linear tri-stapler. The circular stapled anastomosis is routinely oversewn and covered by a pleural tent. Finally, an omental wrap is placed around the anastomosis. At the end of the operation, patients were generally extubated in the operating room and the nasogastric tube was removed. The postoperative care was a standardized procedure for each and every patient. During the study period, the location of the anastomosis on the anterior or posterior wall of the gastric conduit was freely decided by the performing surgeon.

### Differences in reconstruction between RAMIE and MIE

In our center, the intrathoracic anastomosis is performed standardized and quite comparable during MIE and RAMIE, and the only detailed difference is found in the gastric tube forming during the abdominal phase of the operation, which is performed with the robotically linear stapler in the RAMIE procedure and with a common laparoscopic linear stapler in the MIE procedure. Further, the purse string suture is performed with the robotic system in the RAMIE procedure and with laparoscopic hand-suture in MIE procedure. There was no patient selection and the only factor that affected the decision on MIE or RAMIE was the availability of the robotic assistant at the day of the operation.

## Results

**Fig. 3:**
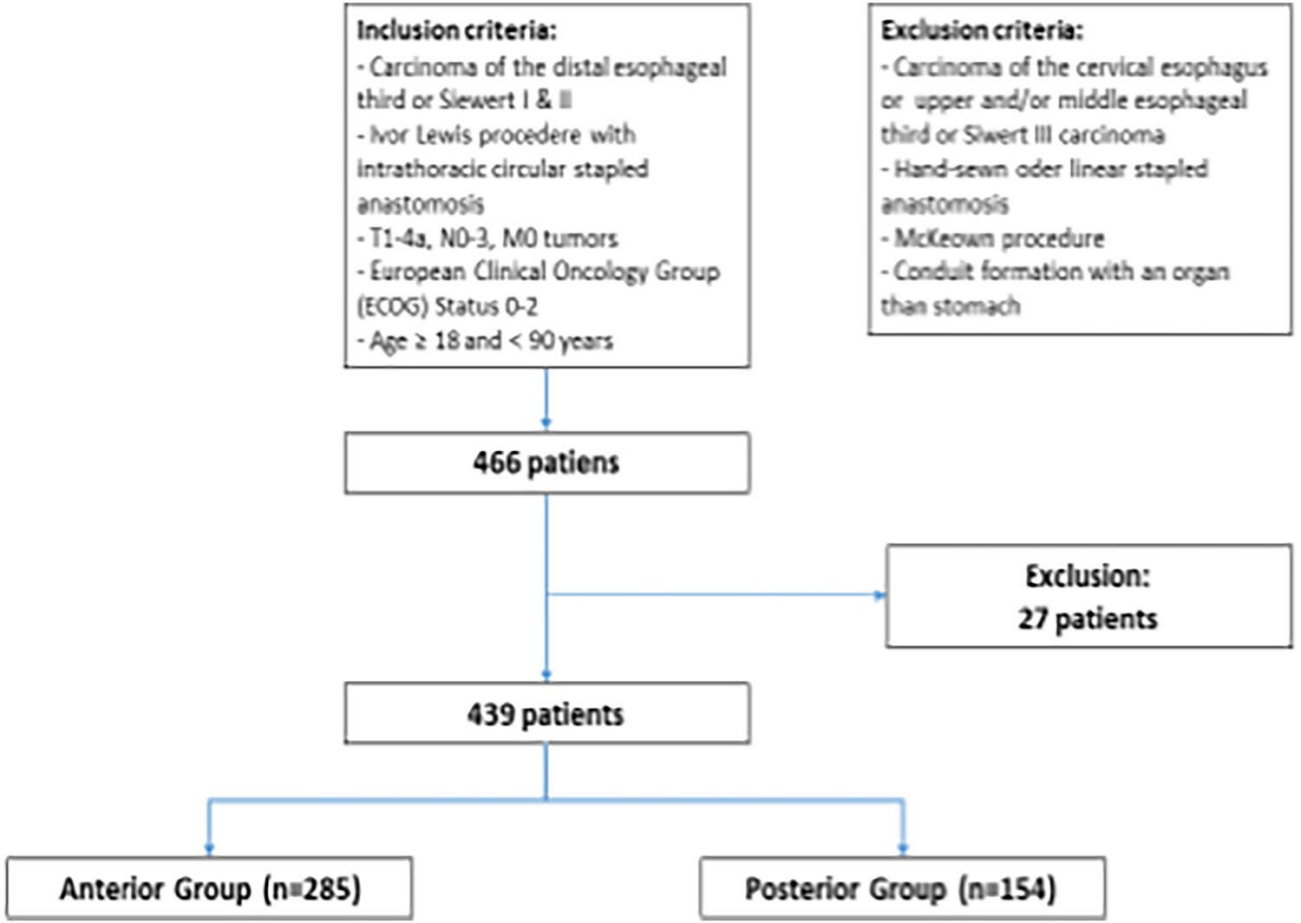
Flowchart for this study

A total of 439 patients were enrolled in the study, 285 (65%) patients in the ANT group and 154 (35%) patients in the POST group. Detailed information in the Flowchart (Fig. 3) Baseline characteristics are summarized in Table 1. No significant differences were observed among demographic characteristics, patients’ comorbidities, the American Society of Anesthesiologists (ASA) score, and tumor location. Regarding tumor type, there was a higher proportion of squamous cell carcinoma in ANT (22.8% vs 16.2%, *P* = 0.043) compared to the POST group. A non-significant higher percentage of patients in the POST did not receive any neoadjuvant treatment compared to ANT (25.3% vs. 20%, *P* = 0.198). Table 2 presents an overview of operative data. Regarding the operative approach, MIE and RAMIE were almost equally performed in POST (50.6% vs. 49.4%), whereas in ANT group, RAMIE was more prevalent compared to MIE (71.2% vs. 28.8%). A conversion to laparotomy was required in two cases (0.7%), both in ANT. No conversion during the thoracic phase was needed. Further, no conversion was performed in POST.

**Table 1:** Patient demographics and tumor characteristics (*n* = 439)

	Anterior (*n* = 285)	Posterior (*n* = 154)	*P*-value
Age (y) (median)(minimum–maximum)	65 (25–86)	64 (29–83)	0.948
Gender (*n* (%))			
Male	234 (82.1)	135 (87.7)	0.238
Female	50 (17.5)	18 (11.7)	
BMI (kg/m^2^) (median)(minimum–maximum)	25 (15–46)	26 (13–47)	0.114
Co-morbidity (*n* (%))			
No comorbidity	72 (25.3)	36 (23.4)	0.662
Vascular	144 (50.5)	89 (57.8)	0.145
Cardiac	61 (21.4)	37 (24.0)	0.529
Diabetes	43 (15.1)	25 (16.2)	0.752
Pulmonary	42 (14.7)	23 (14.9)	0.956
Oncologic	25 (8.8)	18 (11.7)	0.327
Previous abdominal operation	80 (28)	39 (25)	0.537
Neurologic	23 (8.1)	18 (11.7)	0.214
ASA score (*n* (%))			
I	0 (0.0)	1 (0.6)	0.843
II	130 (45.6)	70 (45.5)	
III	142 (49.8)	76 (49.4)	
IV	13 (4.6)	7 (4.5)	
Tumor location (*n* (%))			0.069
Upper esophageal	0 (0.0)	0 (0.0)	
Middle esophageal	24 (8.4)	5 (3.2)	
Lower esophageal	43 (15.1)	20 (13.0)	
GEJ	218 (75.5)	129 (83.7)	
Tumor type (*n* (%))			0.043
Adenocarcinoma	213 (74.7)	128 (83.1)	
Squamous cell carcinoma	65 (22.8)	25 (16.2)	
Melanoma	1 (0.4)	0 (0)	
Neuro-endocrine	5 (1.8)	0 (0.0)	
Neoadjuvant treatment (*n* (%))			0.198
No therapy	57 (20.0)	39 (25.3)	
Chemotherapy	105 (36.8)	82 (53.2)	
Chemoradiotherapy	122 (42.8)	32 (20.8)	
Radiotherapy	1 (0.4)	1 (0.6)	
pT Stage (*n* (%))			0.211
pT0 (complete response)	50 (17.5)	29 (18.8)	
pT1a	16 (5.6)	11 (7.1)	
pT1b	39 (13.7)	25 (16.2)	
pT2	38 (13.3)	23 (14.9)	
pT3	132 (46.3)	62 (40.3)	
pT4a	9 (3.2)	2 (1.3)	
pT4b	1 (0.4)	2 (1.3)	
pN Stage (*n* (%))			0.678
pN0	146 (51.2)	85 (55.2)	
pN1	55 (19.3)	23 (14.9)	
pN2	44 (15.4)	22 (14.3)	
pN3	40 (14)	24 (15.6)	

*ASA score* American society of anesthesiologists physical status classification, *I* patient is a completely healthy fit patient, *II* patient has mild systemic disease, *III* patient has severe systemic disease that is not incapacitating, *IV* patient has incapacitating disease that is a constant threat to life, *GEJ* gastroesophageal junction

**Table 2:** Operative details (*n* = 439)

	Anterior (*n* = 285)	Posterior (*n* = 154)	*P*-value
Operative approach			< 0.001
MIE	82 (28.8)	78 (50.6)	
RAMIE	203 (71.2)	76 (49.4)	
Operating time (min) (median)(minimum–maximum)			
Total operating time	397 ± 79	384 ± 82	0.134
Intraoperative complication	3 (1.1)	0 (0.0)	0.202
Intraoperative conversion			0.297
Abdominal phase	2 (0.7)	0 (0.0)	
Thoracic phase	0 (0.0)	0 (0.0)	
R0 resection	271 (95)	147 (95)	0.864
Blood loss (mean)(minimum–maximum)	158.98 (50–530)	180.7 (10–780)	0.057
No. of lymph nodes (median)(minimum–maximum)	32 (8–81)	33 (7–78)	0.330

*MIE* minimal invasive esophagectomy, *RAMIE* robot-assisted minimal invasive esophagectomy

Postoperative outcomes are illustrated in Table 3. Median length of stay, ICU length of stay, ICU readmission, 30-day, and 90-day mortality did not show any statistical difference between the two groups. Overall complication rate was higher in POST compared to ANT (53.2% vs. 40%, *p* = 0.008), as well as CD > 3 complication rate (36.9% vs. 25.7% *p* = 0.14). Focusing on the primary outcome, anastomotic leakage rate was similar between both groups: 28 (9.8%) cases of anastomotic leakage were detected in ANT and 16 (10.4%) in POST (*p* = 0.851). Delayed gastric emptying, on the other hand, was significantly more frequent in POST compared to ANT (20.1% vs. 7.4% *p* < 0.001). Nosocomial pneumonia rate was 22.1% and 14.8% (*p* = 0.05) in POST and ANT, respectively. From a bivariate analysis emerged that Delayed gastric emptying (DGE) was significantly more frequent after MIE than after RAMIE (18.35% vs. 8.24%, *p* < 0.001) (Table 4). In single groups analysis, analogue results were found in ANT (15.9% vs. 3.9%, *p* = 0.001), whereas no difference was pointed out in POST (20.5% vs. 19.7%, *p* 0.905). Hospital readmission had a higher prevalence in ANT compared to POST (15.4% vs. 8.4% *p* = 0.0038).

**Table 3:** Postoperative data (*n* = 439)

	Anterior (*n* = 285)	Posterior (*n* = 154)	*P*-value
Length of ICU stay (day) (median)(minimum–maximum)	1 (1–115)	1 (1–117)	0.389
Overall complication	114 (40.0)	82 (53.2)	0.008
CD > 3	73 (25.7)	57 (36.9)	
Nosocomial pneumonia (*n* (%))	42 (14.8)	34 (22.1)	0.05
Cardiac complications (*n* (%))	22 (7.7)	15 (9.7)	0.290
Atrial fibrillation	18 (6.3)	5 (3.2)	0.169
Anastomotic leakage (*n* (%))	28 (9.8)	16 (10.4)	0.851
Delayed gastric conduit emptying (*n* (%))	21 (7.4)	31 (20.1)	< 0.001
Chylothorax (*n* (%))	11 (4)	5 (3)	0.744
Recurrent laryngeal nerve paralysis (*n* (%))	9 (3.2)	4 (2.6)	0.741
Wound infection (*n* (%))	6 (2.1)	1 (0.6)	0.246
Hospital stay (day) (median) (minimum–maximum)	12 (7–115)	11 (5–132)	0.888
Readmission 30-day rate (*n* (%))	44 (15.4)	13 (8.4)	0.038
Readmission ICU (*n* (%))	27 (9.5)	17 (11)	0.603
30-day mortality	4 (1.4)	2 (1.3)	0.928
90-day mortality	10 (3.5)	2 (1.3)	0.176

*CD* Clavien–Dindo classification [12]

**Table 4:** Delayed gastric emptying (DGE) associated to the operative procedure

	Delayed gastric emptying	*P*-value
Entire cohort	52 (11.84)	< 0.001
MIE	29 (18.35%)	
RAMIE	23 (8.24%)	
POST group		0.905
MIE	16 (20.5%)	
RAMIE	15 (19.7%)	
ANT group		0.001
MIE	13 (15.9%)	
RAMIE	8 (3.9%)	

*MIE* minimal invasive esophagectomy, *RAMIE* robot-assisted minimal invasive esophagectomy, *ANT group* anterior gastric wall anastomosis group, *POST group* posterior gastric wall anastomosis group

## Discussion

In circular stapled esophagogastric anastomosis, one core step is the selection of the proper location for the anastomosis. In our department, we chose the specific side of the gastric conduit, according to the best anatomic suiting position for the anastomosis. Whether this step could affect AL rate has not been investigated yet. Thus, we decided to compare our patients who received an esophagogastric anastomosis on the anterior wall to those who received the anastomosis on the posterior wall. Additionally, the same surgical team, using the same standardized technique, as well as preoperative work-up and postoperative care was carried out for each operation. Our data suggest that the location of the esophagogastric anastomosis in TMIIL does not affect the rate of anastomotic leakage.

Interestingly, we observed that overall complication rate and CD > 3 complication rate were significantly higher in POST compared to ANT (53.2% vs. 40% (*p* = 0.008) and 36.9% vs. 25.7% (*p* = 0.014), respectively). Analyzing the specific postoperative complications, we found out a difference between the groups regarding the DGE and nosocomial pneumonia rate; both complications were noticeably higher in POST group. DGE after esophagectomy is a clinically relevant disorder, whose incidence range between 10 and 20% [14, 15]. Moreover, mild symptoms could appear in over 50% of patients [16]. This condition is also associated with severe short-term adverse outcomes, like AL, aspiration pneumonia, and prolonged ICU stay [17]. Causes of DGE are pyloric dysfunction at first, but also torsion or angulation of the conduit, redundant gastric conduit, and insufficient widening of esophageal hiatus [18]. Regarding the possibly most relevant cause of DGE, the pyloric dysfunction, all patients were treated similarly with preoperative endoscopic balloon dilatation, which is a standard protocol in this institution. This protocol has been demonstrated to be more effective than postoperative pyloric treatment on demand [13]. In this sense, all other factors contributing to DGE need to be included in the discussion. Konradson et al. [19] first stated that even the level of the anastomosis and the anastomotic technique could play a role. In the current study, the anastomotic technique is for both MIE and RAMIE procedures are standard and utterly comparable. To further elaborate the latter the width of the conduit, the height of the intrathoracic anastomosis, the preparation of the diaphragmatic crura and anastomotic creation are common for both procedures. The only step that differs is the anterior or posterior placement of the anastomosis which is why it is controlled in our study.

In our series, the overall DGE rate was 11.84%, consistent with the literature data [20]. Single-group percentages, instead, were 7.4% and 20.1%, respectively, in anterior (ANT) and posterior (POST) group (*P* < 0.001). As previously shown, the highest incidence of DGE in POST led to a higher rate of nosocomial pneumonia (22.1% vs. 14.8%, *P* = 0.05). However, median length of stay, ICU length of stay, and ICU readmission were similar in both groups. Surprisingly, 30-day unplanned hospital readmission occurred significantly more often in ANT. This finding seems to be inconsistent with the higher overall and CD > 3 complication rate observed in patients belonging to POST. The overall readmission rate of 12.9% (57/439) however appears acceptable for an oncologic esophageal resection.

As previously pointed out, every operation was performed with the same standardized technique [21, 22], by the same experienced team and we have no reason to believe that other surgical elements, such as the conduit’s length or the esophageal hiatus’ size, could have triggered the difference between ANT and POST group. However, conduit torsion in the posterior anastomosis might cause DGE, and although not apparent in this study, we believe it has to be further investigated in comparative studies. In the anterior group, RAMIE was performed more frequently than MIE. For this reason, we have performed a bivariate analysis (Table 4), considering the operative technique (MIE and RAMIE) and the DGE rate, to define if there was an association between these variables. In the overall cohort, DGE was significantly more frequent after MIE than after RAMIE (18.35% vs. 8.24%, *P* < 0.001). However, when individually analyzing the anterior and the posterior group, analogue results were found in ANT (15.9% vs. 3.9%, *P* = 0.001), whereas no difference could be depicted in POST (20.5% vs. 19.7%, *P* 0.905). The latter results suggest that the location of the anastomosis played the most significant role in regard of DGE. On the other hand, the minimally invasive technique used (MIE vs. RAMIE) affected the general results, thus detailed differences between RAMIE and MIE need to be considered. One explanation might be that the robotic *EndoWrist* stapler with its higher angulation freedom to the hand-held minimally invasive stapler instruments might allow to perform a “straighter” staple line, parallel to the greater curvature, avoiding any sharp angulation or lateral sliding of the suture line on the anterior surface of the stomach. Such “sharp edges” may act as anchoring sites that can produce a partial twist of the conduit and lead to DGE. There were no significant differences in the number of staplers used although the number can vary according to the size of the stomach of each patient. However, this difference has not been seen in POST, in which MIE and RAMIE were almost equally performed, and DGE rate was similar among these subgroups. Furthermore, also considering separately MIE and RAMIE (Table 5), DGE rate remains sensibly higher in POST compared to ANT (20.5% vs. 15.9% and 19.7% vs. 3.8%, respectively, in MIE and RAMIE).

**Table 5:** Delayed gastric emptying (DGE) subgroup analysis

	Delayed gastric emptying		*P*-value
	ANT	POST	
MIE	13 (15.9%)	16 (20.5%)	0.288
RAMIE	8 (3.9%)	15 (19.7%)	< 0.001

*MIE* minimal invasive esophagectomy, *RAMIE* robot-assisted minimal invasive esophagectomy, *ANT group* anterior gastric wall anastomosis group, *POST group* posterior gastric wall anastomosis group

In regards of possible differences between MIE and RAMIE, the ongoing ROBOT-2 trial [23], led by our team, may further clarify the impact of the robot on DGE after IL esophagectomies, giving the statistical power of a multicenter randomized controlled trial.

Limitation of this study is the retrospective design, which naturally can lead to biased results. Whereas, the large cohort of patients consecutively operated by the same surgical team at the same high-volume center represents the strength of the study. Thus, the same surgical technique has been adopted for every step of each operation, included the anastomosis, both performed in a MIE or a RAMIE. Moreover, postoperative patient management was standardized and the same diagnostic criteria for early detection of postoperative complications were adopted for every patient. A randomized controlled trial is need to further evaluate out hypothesis generating study about the optimal location of the intrathoracic circular stapler anastomosis during a minimal invasive Ivor Lewis esophagectomy.

## Conclusion

Due to our findings, we conclude that the specific location of the anastomosis may not be associated with the anastomotic leakage rate. However, the anterior wall of the gastric conduit appears to be the most suitable location for the anastomosis in a TMIIL, especially when performed with a robot-assisted approach, to prevent DGE and nosocomial pneumonia.
